# Application of machine learning in predicting adolescent Internet behavioral addiction

**DOI:** 10.3389/fpsyt.2024.1521051

**Published:** 2025-04-01

**Authors:** Yao Gan, Li Kuang, Xiao-Ming Xu, Ming Ai, Jing-Lan He, Wo Wang, Su Hong, Jian mei Chen, Jun Cao, Qi Zhang

**Affiliations:** ^1^ Department of Psychiatry, The First Affiliated Hospital of Chongqing Medical University, Chongqing, China; ^2^ Mental Health Center, University-Town Hospital of Chongqing Medical University, Chongqing, China

**Keywords:** machine learning, adolescent, internet behavior addiction, risk factors, prediction model

## Abstract

**Objective:**

To explore the risk factors affecting adolescents’ Internet addiction behavior and build a prediction model for adolescents’ Internet addiction behavior based on machine learning algorithms.

**Methods:**

A total of 4461 high school students in Chongqing were selected using stratified cluster sampling, and questionnaires were administered. Based on the presence of Internet addiction behavior, students were categorized into an Internet addiction group (n=1210) and a non-Internet addiction group (n=3115). Gender, age, residence type, and other data were compared between the groups, and independent risk factors for adolescent Internet addiction were analyzed using a logistic regression model. Six methods—multi-level perceptron, random forest, K-nearest neighbor, support vector machine, logistic regression, and extreme gradient boosting—were used to construct the model. The model’s indicators under each algorithm were compared, evaluated with a confusion matrix, and the optimal model was selected.

**Result:**

The proportion of male adolescents, urban household registration, and scores on the family function, planning, action, and cognitive subscales, along with psychoticism, introversion-extroversion, neuroticism, somatization, obsessive-compulsiveness, interpersonal sensitivity, depression, anxiety, hostility, paranoia, and psychosis, were significantly higher in the Internet addiction group than in the non-Internet addiction group (P < 0.05). No significant differences were found in age or only-child status (P > 0.05). Statistically significant variables were analyzed using a logistic regression model, revealing that gender, household registration type, and scores on planning, action, introversion-extroversion, psychoticism, neuroticism, cognitive, obsessive-compulsive, depression, and hostility scales are independent risk factors for adolescent Internet addiction. The area under the curve (AUC) for multi-level perceptron, random forest, K-nearest neighbor, support vector machine, logistic regression, and extreme gradient boosting models were 0.843, 0.817, 0.778, 0.846, 0.847, and 0.836, respectively, with extreme gradient boosting showing the best predictive performance among these models.

**Conclusion:**

The detection rate of Internet addiction is higher in males than in females, and adolescents with impulsive, extroverted, psychotic, neurotic, obsessive, depressive, and hostile traits are more prone to developing Internet addiction. While the overall performance of the machine learning models for predicting adolescent Internet addiction is moderate, the extreme gradient boosting method outperforms others, effectively identifying risk factors and enabling targeted interventions.

## Introduction

Internet addiction refers to a chronic or cyclical dependence that occurs after an individual repeatedly uses the Internet. It is also accompanied by a series of addictive symptoms and pathological or deviant Internet usage behaviors, including Internet game addiction, Internet shopping addiction, Internet gambling addiction, Internet pornography addiction, among which Internet game addiction is particularly common (1–3). With the widespread use of the Internet, the detection rate of Internet addiction among teenagers is increasing year by year, and it has become a serious personal, family and social problem (4). Internet addiction can cause adolescents to suffer from reduced sleep quality, weakened physical constitution, anxiety and depression, multiple personality disorders and social maladaptation, etc., seriously damaging the psychological and social functions of adolescents (5). Internet addiction can bring many negative effects, and it can induce young people to commit crimes such as rape, theft, fraud, and prostitution, seriously affecting social stability (6). A systematic analysis of Internet addiction risk factors found that Internet addicted teenagers have a higher risk of suicidal behavior, drinking and smoking (7). Therefore, it is particularly important to explore effective methods for early identification of adolescent Internet addiction and provide timely intervention. Machine learning is an efficient and automated process method that can be used to judge and predict specific content in the real world. Its essence is to use computer systems to explore patterns in large-scale data through some algorithms and statistical models, simultaneously using models that can recognize this pattern to describe or predict new data (8–11). At present, there are few research reports on machine learning in adolescent Internet addiction behavior. Based on the above background, this study builds a machine learning model of adolescent Internet addiction behavior and analyzes the risk factors that affect adolescent Internet addiction behavior, to provide more possibilities for predicting internet addiction behavior.

## 2 Materials and methods

### 2.1 Normal information

4461 high school students in Chongqing were selected as the research subjects, stratified cluster sampling was used, and questionnaires were distributed. After excluding questionnaires with obvious logical errors, a total of 4,325 questionnaires were recovered, with an effective response rate of 96.96%. All research subjects agreed to participate in this study and signed an informed consent form. All parents of minors were informed and signed an informed consent form.

### 2.2 Method

#### 2.2.1 Grouping and standards

The Internet addiction behavior of adolescents is evaluated through the Internet Addiction Scale compiled by Young, which includes time management, Internet addiction tolerance, compulsive Internet use, Internet addiction withdrawal reaction, etc., in summary Divided into 20~100 points. Among them, adolescents with a score of <40 points were included in the non-Internet addiction group, adolescents with a score of ≥40 were included in the Internet addiction group. 60 points, 80 points, and 100 points serve as the dividing points for mild, moderate, and severe internet addiction, respectively. The Cronbach alpha system of the Internet Addiction Scale is 0.87, and the 95% CI is (0.84~0.90); the estimated validity coefficient is 0.72, and the 95% CI is (0.64~0.75).

#### 2.2.2 Data collection

(1) General data: data on sex, age, registered residence type, whether or not an only child, family function, etc. of adolescents in the Internet addiction group and non-Internet addiction group were collected. (2) Impulsive personality traits: The Barratt Impulsiveness Scale Chinese version (BIS-CV) was used to evaluate the impulsive personality traits of the two groups of adolescents, including behavioral, cognitive and unplanned impulsivity. The higher the score, the stronger the impulsiveness. (3) Mental and psychological status: The mental and psychological status of the two groups of adolescents was assessed through the Chinese version of the Symptom Self-rating Scale (SCL-90), including somatization, obsessive-compulsiveness, interpersonal sensitivity, depression, anxiety, hostility, hostility, Paranoid, psychotic and other 10 factors, 90 items, are used to test the general symptoms and psychological problems of adolescents in the past 7 days. If an item scores <2 points, it is considered a positive item. (4) Personality tendencies and characteristics: The Eysenck Personality Questionnaire (EPQ) was used to evaluate the personality tendencies and characteristics of the two groups of adolescents, including the three dimensions of introversion-extroversion (E), neuroticism (P) and psychoticism (N). The score adopts the form of T score, which evaluates the personality tendencies and characteristics of the research subjects according to the score. Scores of 38.5~43.4 and 56.7~61.5 are considered prone types, 43.5~56.7 are considered intermediate types, and <38.5 and >61.5 are considered typical types.

#### 2.2.3 Build a model

Construct a classification prediction model for Internet addiction behavior based on the general information, personality and psychological characteristics of the research object. The first step is to determine whether the target behavior belongs to Internet addiction behavior, and further set a two-category label, where 0=none, 1= have. Integrate all adolescent information and build a classification prediction model for Internet addiction behavior. Model construction was carried out through six methods: multi-level perceptron, random forest, K-neighbor algorithm, support vector machine, logistic regression and extreme gradient boosting method. The indicators of the suicide and self-injury behavior model under the six algorithms were compared, and the best one was further selected.

### 2.3 Observation indicators

(1) Compare the gender, age, household registration type and other general information, personality and psychological characteristics of adolescents in the Internet addiction group and the non-Internet addiction group. (2) Analyze the independent risk factors that influence adolescents’ Internet addiction behaviors. (3) Construct different early warning models for adolescent Internet addiction behavior and analyze their performance. (4) Use confusion matrices to evaluate the construction results of different early warning models for adolescent Internet addiction behavior.

### 2.4 Statistical methods

The research data were analyzed by SPSS 21.0, and the counting data were expressed by [n(%)], and the *x* ^2^ test was used for pairwise comparison. The measurement data that conform to the normal distribution and homogeneity of variance are expressed by ( $\bar{x}$ ± *s)*, and the pairwise comparison passes the independent sample t test; Logistic regression model was used to analyze the independent risk factors affecting adolescents’ Internet addiction behavior. The results of model construction are evaluated by confusion matrix. *P* < 0.05 was considered as a significant difference.

## 3 Results

### 3.1 Single factor analysis on the relationship between adolescents and Internet addiction behaviors

Proportion of male adolescents in the Internet addiction group, proportion of urban household registration, general proportion of family functions and planning subscale, action subscale, cognitive subscale, psychoticism, introversion-extroversion, neuroticism, somatization, obsessive - compulsiveness, interpersonal sensitivity, depression, anxiety, hostility, hostility, paranoia, psychosis and other scores were significantly increased compared with the non-Internet addiction group (*P <*0.05). There was no statistically significant difference in age and type of only child among adolescents in the internet addiction group (*P* > 0.05). See **Table 1**.

**Table 1:** Univariate analysis of the relationship between adolescents and Internet addictive behaviors [n (%), ( $\bar{x}$ ± *s)*].

parameter	internet addiction group (n=1210)	Non-Internet addiction group (n=3115)	*x2/t* ^_^	*P*
gender			16.271	0.000
male	704 (58.18)	1600 (51.36)		
female	506 (41.82)	1515 (48.64)		
age)			1.177	0.278
≥15	615 (50.83)	1526 (48.99)		
<15	595 (49.17)	1589 (51.01)		
Household registration type			8.089	0.004
town	701 (57.93)	1643 (52.74)		
rural area	509 (42.07)	1427 (47.26)		
only child			3.644	0.056
yes	628 (51.90)	1516 (48.67)		
no	582 (48.10)	1599 (51.33)		
family function			27.422	0.000
better	654 (54.05)	1954 (62.73)		
generally	556 (45.95)	1161 (37.27)		
planning subscale	52.65 ± 15.25	42.13 ± 13.04	21.175	0.000
action subscale	39.90 ± 12.26	27.60 ± 8.22	32.167	0.000
cognitive subscale	50.16 ± 13.65	44.78 ± 9.69	12.536	0.000
Psychopathy	60.78 ± 11.81	53.24 ± 10.82	19.318	0.000
introversion-extroversion	48.97 ± 10.87	49.44 ± 10.18	1.348	0.178
neurotic	59.49 ± 10.37	51.68 ± 10.46	22.062	0.000
Somatization	18.84 ± 3.22	15.01 ± 2.27	37.768	0.000
force	21.90 ± 5.23	17.59 ± 3.23	26.691	0.000
interpersonal sensitivity	18.40 ± 3.35	13.54 ± 2.25	46.294	0.000
depression	24.49 ± 6.66	18.29 ± 4.41	29.848	0.000
anxiety	18.44 ± 3.74	13.79 ± 3.50	37.321	0.000
hostility	11.81 ± 2.23	8.47 ± 1.93	45.410	0.000
Paranoid	11.55 ± 2.15	8.22 ± 1.56	48.882	0.000
Psychotic	17.77 ± 4.12	13.34 ± 2.15	35.264	0.000
other	12.62 ± 3.37	9.37 ± 2.55	30193	0.000

### 3.2 Logistic multi-factor analysis on the relationship between adolescents and Internet addiction behaviors

Statistically significant parameters such as gender, household registration type, family function, planning subscale, action subscale, and cognitive subscale were included in the Logistic risk regression model for analysis. The results showed that gender, household registration type, planning subscale, and action subscale Table, introversion-extroversion, psychoticism, neuroticism, cognitive subscale, obsessive-compulsiveness, depression and hostility scores can all be used as independent risk factors for adolescents to develop Internet addiction behaviors. See **Table 2**.

**Table 2:** Logistic multifactor analysis of adolescents’ relationship with Internet addiction behaviors.

parameter	*β*	*SE*	*Wald*	*P*	*OR*	95%CI
gender	0.58 2	0.310	6.056	0.041	1.789	1.658~2.698
Household registration type	0.50 2	0.256	8.881	0.001	1.652	1.023~1.956
family function	0.12 8	0.168	4.535	0.087	1.136	0.986~1.562
planning subscale	0.75 4	0.305	8.105	0.003	2.125	1.569~3.154
action subscale	0.68 6	0.256	10.468	0.000	1.985	1.459~2.123
cognitive subscale	0.63 5	0.277	9.043	0.000	1.887	1.698~1.996
Eysenck personality standard score P	0.22 6	0.155	9.407	0.000	1.253	0.854~1.286
Eysenck personality standard score E	0.097	0.105	8.798	0.001	1.102	0.569~1.236
Eysenck personality standard score N	0.42 1	0.251	6.682	0.037	1.523	0.965~1.695
force	0.75 3	0.352	6.077	0.041	2.123	1.854~4.156
interpersonal sensitivity	0.02 3	0.075	4.089	0.101	1.023	0.965~1.196
depression	0.450	0.262	6.556	0.039	1.569	1.325~1.965
anxiety	0.105	0.158	4.206	0.085	1.111	0.754~1.695
hostility	0.46 8	0.222	9.496	0.000	1.596	1.459~2.125
Paranoid	0.035	0.235	3.839	0.162	1.036	0.569~1.966
Psychotic	0.173	0.195	4.550	0.088	1.189	0.965~1.269
other	0.21 2	0.221	4.341	0.082	1.236	0.652~1.365

### 3.3 Analysis of early warning models of adolescent Internet addiction behavior in different models

**Figure 1:**
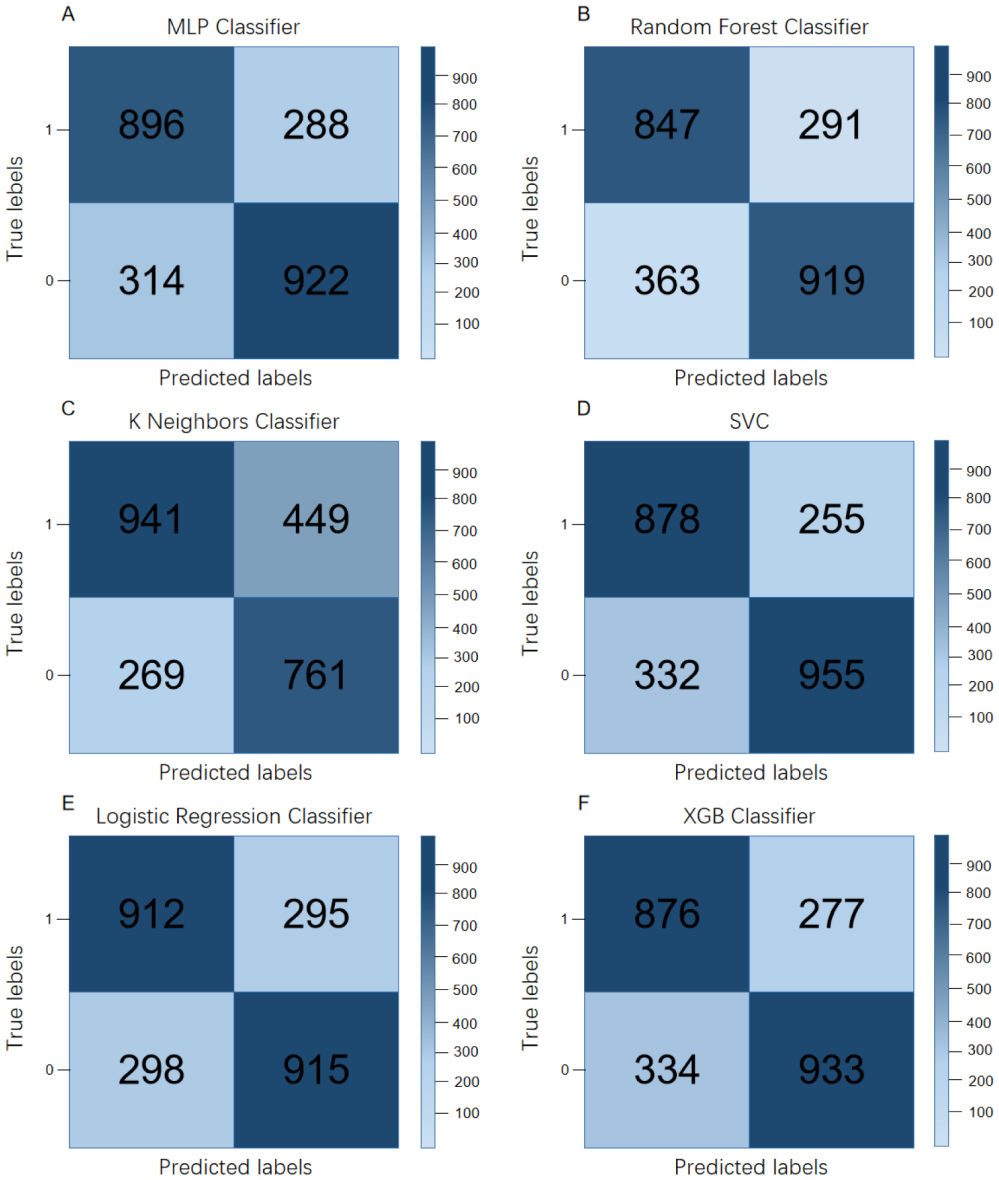
Confusion matrix of different models for early warning of adolescent internet addiction behavior. **(A)** represents the multilevel perceptron classification method; **(B)** represents the random forest classification method; **(C)** represents the K neighbor algorithm classification method; **(D)** represents the support direction classification method; **(E)** represents the logistic regression; **(F)** represents the extreme gradient boosting classification method.

The area under the curve (AUC) of the multi-level perceptron, random forest, K-neighbor algorithm, support vector machine, logistic regression and extreme gradient boosting early warning model are 0.843, 0.817, 0.778, 0846, 0.847, 0.836 respectively. The performance in predicting adolescent internet addiction behavior is average, with the extreme gradient enhancement method performing better than other models. See **Figure 1**, **Table 3**.

**Table 3:** Performance analysis of adolescent internet addiction behavior warning models with different models.

Model Type	Sensitivity	Specificity	Positive Predictive Value	Negative Predictive Value	Accuracy	AUC
multi-level perceptron	0.7718	0.7516	0.7568	0.7669	0.7617	0.843
random forest	0.7603	0.7114	0.7281	0.7552	0.7409	0.817
K-Nearest Algorithm	0.6399	0.7892	0.7502	0.6881	0.7145	0.778
Support Vector Machines	0.8003	0.7373	0.7535	0.7861	0.7688	0.846
logistic regression	0.7675	0.7656	0.7560	0.7670	0.7665	0.847
extreme gradient boosting	0.7820	0.7356	0.7478	0.7708	0.7588	0.836

## 4 Discussion

Internet addiction refers to the decline in academic, work, and physical and mental functions caused by excessive use of the Internet. With the vigorous development of the Internet, it has become an important tool for teenagers. However, the excessive use of the Internet has gradually increased the number of Internet addictive behaviors (12–14). The current domestic research reports on adolescent Internet addiction behavior have a small sample size and insufficient attention, which may lead to repeated screening and missed selection, resulting in the inability to early identify adolescent Internet addiction behavior and achieve Timely and effective intervention.

Proportion of male adolescents in the Internet addiction group, proportion of urban household registration, general proportion of family functions and planning subscale, action subscale, cognitive subscale, psychoticism, introversion-extroversion, neuroticism, somatization, obsessive - compulsiveness, interpersonal sensitivity, depression, anxiety, hostility, hostility, paranoia, psychosis and other scores were significantly increased compared with the non-Internet addiction group (*P <*0.05). Statistically significant parameters were included in the Logistic risk regression model for analysis. The results showed that gender, registered residence type, planning subscale, action subscale, introversion extroversion, psychoticism, neuroticism, cognitive subscale, obsessive compulsion, depression and hostility scores were all independent risk factors for adolescents to develop Internet addiction. Gender can be a risk factor affecting adolescents’ Internet addiction behavior. Wu (15) and Wang (16) and others found that boys like excitement and adventure, lack effective emotional expression, and are not good at using methods such as crying and talking to relieve depression. They have poor self-control, so they will adopt a negative coping style when encountering difficulties, and are more likely to turn to virtual networks for comfort. Teenagers whose household registration is in urban areas have better economic conditions and good network communication conditions. Compared with rural teenagers, they can access network systems earlier. In addition, the relative limitations of urban living and entertainment spaces have increased the dependence of teenagers on computers, mobile phones, and other devices, thereby increasing their reliance on the internet for social interaction and stress relief (17, 18). The gradual formation of personality is closely related to the occurrence and progression of adolescent Internet addiction. Impulsiveness and aggressive personality are both risk factors for Internet addiction. Teenagers with impulsive and aggressive personalities prefer to relieve stress through simple and fast methods. In reality, they lack the ability to communicate with others and tend to obtain pleasure from the internet; these teenagers are also affected by violent online games, short and fast messages, etc., leading to changes in the way of thinking, emotional instability, easy to act impulsively and imitate online some simple and irrational behaviors, eventually lead to a vicious circle (19, 20). For adolescents, their addictive behaviors and impulsive behaviors may have a common origin in the frontostriatal pathway in neurobiological mechanisms (21, 22). Extraversion, high psychoticism and high neuroticism are important traits of people susceptible to Internet addiction. However, the current research on personality introversion-extroversion has not been completely consistent. If any research has found that most teenagers with low extraversion have Real social barriers, online socialization is asynchronous and anonymous, and they can obtain social compensation in online socialization (23). Other scholars have shown that adolescents with high extraversion are at greater risk of becoming addicted to the Internet due to curiosity and excitement, or to enhance social interaction (16). People with high neuroticism are emotionally unstable and need to alleviate or transfer bad emotions in certain ways, and the Internet is a convenient way. Communicating with others through the Internet can effectively reduce fear and improve mood swings (24, 25).

Machine learning has long been an important part of computer science, and in recent years it has been gradually applied to clinical psychology. Machine learning can assist in diagnosis, disease classification prediction, medical image recognition, etc. It is an important tool that can be used to judge and predict specific content in the real world. It can train datasets with multiple features, and then select appropriate models by clarifying the possible set of models. After clarifying the learning strategy, the optimal model algorithm is used to analyze the data, which plays a crucial role in predicting the occurrence of health risk behaviors (26–28). Compared with traditional methods, machine learning can provide people with more models for building classification and prediction of risky health behavior patterns (29). Ahn (30) et al. used support vector machines to analyze the underlying neural mechanisms of Internet addiction behavior and other mental diseases based on the fMRI imaging data of Internet addicted individuals, and conducted machine learning on the psychological and personality characteristics of college students’ Internet addiction behavior. Learning analytics can be used to predict potentially risky health behaviors.

The results of this study show that the area under the curve (AUC) of the multi-level perceptron, random forest, K neighbor algorithm, support vector machine, logistic regression and extreme gradient boosting early warning model are 0.843, 0.817, 0.778, 0846, 0.847, 0.836 respectively. The performance in predicting adolescent internet addiction behavior is average, with the extreme gradient enhancement method performing better than other models.

However, this survey analysis has certain limitations: (1) The relevant data and content obtained during the survey are only derived from cross-sectional data and are not analyzed from the perspective of cohort studies or prospective studies. Group effects may occur or key points in the development process may be overlooked; (2) The survey on adolescents only analyzed their gender, age, family and other general information and related social and psychological factors, and did not include factors such as imaging and electrophysiology, which may lead to a certain bias in the results. (3) Survey analysis conducted through adolescent self-reporting shows that in order to avoid shame and self-exposure, some adolescents may conceal risky behaviors. There is also retrospective bias, and the accuracy needs to be examined. (4) This study aims to build an ideal Internet addiction prediction model, and the performance of models constructed using different methods is similar.

To sum up, the detection rate of Internet addictive behavior in men is higher than that in women, and adolescents with impulsive, extroverted, psychotic, neurotic, obsessive, depressive and hostile traits are more likely to develop Internet addictive behavior. The performance of the classification prediction model for adolescent Internet addiction behavior built using machine learning is average, but the extreme gradient boosting method performs better than other models. It can identify adolescent risk factors to a certain extent and make targeted interventions.

## Data Availability

The raw data supporting the conclusions of this article will be made available by the authors, without undue reservation.
